# Editorial: Beneficial elements: novel players in plant biology for innovative crop production, volume II

**DOI:** 10.3389/fpls.2023.1303462

**Published:** 2023-10-18

**Authors:** Libia Iris Trejo-Téllez, Fernando Carlos Gómez-Merino

**Affiliations:** ^1^ Department of Soil Sciences, Laboratory of Plant Nutrition, College of Postgraduates in Agricultural Sciences, Texcoco, Mexico, Mexico; ^2^ Cooperative Research Group at the Department Agri-Food Sustainable Innovation, College of Postgraduates in Agricultural Sciences, Amatlán de los Reyes, Veracruz, Mexico; ^3^ Department Agri-Food Sustainable Innovation, Cooperative Research Group at College of Postgraduates in Agricultural Sciences, Veracruz, Mexico

**Keywords:** biostimulation, inorganic biostimulants, rare earth elements, lanthanides, agricultural productivity, climate change, abiotic stress tolerance

## 1 Introduction

In this Research Topic entitled “*Beneficial Elements: Novel Players in Plant Biology for Innovative Crop Production*”, we aimed to expand our knowledge about the responses of plants to the application of beneficial elements as inorganic biostimulants to boost their growth and development, cope with salt stress, and enhance productivity and quality traits at harvest. This Research Topic contains five scientific articles (all original research articles) that contribute to a better understanding of the processes that can be induced in plants exposed to different beneficial elements applied as inorganic biostimulants: sodium, silicon and titanium. Interestingly, authors contributing to this Research Topic have affiliations with institutions in different countries, including China, Czechia, Hungary, India, Israel, Mexico, Serbia, Slovakia, and the USA. This Foreword was written to contextualize the importance of these elements and to summarize the valuable papers published in this Research Topic in order to lead to a wider visibility and citation of these studies.

## 2 Beneficial elements as potent inorganic biostimulants

From a biological sciences perspective, biostimulation is a natural phenomenon present in all biological systems. This phenomenon involves the modification of the environmental conditions in which an organism thrives. Such a modification is aimed at eliciting or strengthening optimal responses to an external stimulus in terms of structures and behaviors (Adams et al., 2015; Myazin et al., 2021) that make them well-adapted to tackle challenging environments. Changes in the environment may result in negative, neutral or positive responses of individuals within a population (Keenan, 2020), and those changes may be triggered by stimuli of a physical, chemical or biological nature (Hunt et al., 2009). Currently, biostimulation principles are gaining momentum in plant science and especially in sustainable agriculture approaches (Garza-Alonso et al., 2022; Gupta et al., 2023).

Although a consensual definition and the scope of a plant biostimulant are still being discussed, it is accepted that this technical term includes beneficial substances, microorganisms, or both, whose function when applied to plants or to soil is to boost natural processes to enhance or benefit physiological, biochemical and molecular mechanisms in terms of growth and development; nutrient uptake and nutrient use efficiency; tolerance to abiotic stress; and crop yield and quality (de Vasconcelos and Chaves, 2019; Rouphael and Colla, 2020; Juárez-Maldonado et al., 2021; Garza-Alonso et al., 2022). Plant biostimulants may be chemical or biological in origin, and the chemicals may be natural or synthetic, organic or inorganic (Colla and Rouphael, 2015; du Jardin, 2015). Currently, seven categories of plant biostimulants are well accepted: 1) humic substances; 2) protein hydrolysates and other N-containing compounds; 3) seaweed extracts and botanicals; 4) chitosan and other biopolymers; 5) inorganic compounds; 6) beneficial fungi; and 7) beneficial bacteria.

Among biostimulants, inorganic compounds comprise phosphite salts and beneficial elements. Beneficial elements are defined as the mineral elements that stimulate plant growth and have beneficial effects, especially at very low concentrations (Kaur et al., 2016; Lo Piccolo et al., 2021; Ceccanti et al., 2023). Unlike the macronutrients nitrogen (N), phosphorus (P), potassium (K), calcium (Ca), magnesium (Mg) and sulfur (S), and the micronutrients chlorine (Cl), boron (B), iron (Fe), manganese (Mn), zinc (Zn), copper (Cu), nickel (Ni) and molybdenum (Mo), beneficial elements are not essential for all plant species, but have been found to affect the uptake, translocation and utilization of other essential elements, help in the synthesis of vital metabolites and also counteract the toxic effects of some other toxic elements or anti metabolites (Kirkby, 2023). So far, we have defined ten beneficial elements that may act as inorganic biostimulants: aluminum (Al), cerium (Ce), cobalt (Co), iodine (I), lanthanum (La), sodium (Na), selenium (Se), silicon (Si), titanium (Ti), and vanadium (V) (Gómez-Merino and Trejo-Téllez, 2018; Trejo-Téllez et al., 2023). These elements emerging as novel biostimulants may enhance crop productivity and nutritional quality while improving responses to environmental stimuli and stressors in some plant species (Rouphael et al., 2021). When supplied at low dosages, they help improve plant growth, development, and yield quality by stimulating different molecular, biochemical, and physiological mechanisms triggering adaptive responses to challenging environments.

## 3 Beneficial elements as novel players in plant biology for innovative crop production

So far, the following stimulant effects of beneficial elements have been demonstrated, which have been reviewed by Gómez-Merino and Trejo-Téllez (2018) and Trejo-Téllez et al. (2023). Aluminum (Al) can modulate the color of flowers, stimulate plant growth and root development, increase the vase life of some species and trigger antioxidant mechanisms. Cerium (Ce) may increase root size and the number of root hairs boosts catalase activity and may be involved in the transformation of inorganic N to organic N. In legumes, cobalt (Co) plays a major role in atmospheric N fixation by *Rhizobium*, and in other species, it can improve the use of P, K, Mn, and Zn, as well as increase vase life. Iodine (I) can improve the use of N, advance flowering, and increase fruit yield and uniformity, while I biofortification approaches in crop plants are widely recognized. Lanthanum (La) can improve uptake of K, Ca, and Mg, increase root and overall plant growth, improve germination processes, participate in signaling processes mediated by Ca-calmodulin and activate antioxidant mechanisms. In biofortification assays, selenium (Se) may improve tolerance to oxidative stress, reduce the senescence process, promote growth, and increase the uptake of heavy metals. Silicon (Si) can counteract the deleterious effects of contaminants, drought, and salinity, induce resistance mechanisms against pests and diseases, allow the formation of nanostructures, improve the strength and stiffness of plant tissues, stimulate antioxidant mechanisms, reduce ethylene synthesis, and increase vase life. Sodium (Na) can act as a growth regulator, improve vase life and stimulate the synthesis of amino acids such as proline. Titanium (Ti) improves uptake of N, P, K, Ca, and Mg, increases starch synthesis, and generally improves plant growth. Vanadium (V) can act as an inducer of secondary metabolism and increase plant growth. This list may be expanded, and elements such as silver (Ag), chromium (Cr), fluorine (F), tungsten or wolframium (W), and various lanthanides may also have beneficial effects on plant biology, although they have been little explored. Indeed, we recently demonstrated that neodymium (Nd) promotes growth, nutrient concentration, and metabolism in sugarcane (Ramírez-Antonio et al., 2023). Beneficial elements, then, have great potential for use in facing some of the most daunting challenges facing humanity, such as climate change and growing food demand. Therefore, further research on beneficial elements is crucial for global agri-food innovation.

Among the ten cited beneficial elements recognized to date, in this Research Topic authors have approached the effects of sodium (Na), silicon (Si) and titanium (Ti) on different attributes of the following plant species: Arabidopsis, rapeseed, potato, rice, and pedunculate oak. In the next paragraphs, we present a summary of the main findings reported by different research groups in this Research Topic.

In potato, Han et al. reported that tubers exposed to 50 mM sodium silicate accelerated the formation of wound healing structures and significantly reduced the weight loss and disease index of tubers. This source of Si induced the gene expression and enzyme activity of phenylalanine ammonia lyase (PAL), 4-coumarate: coenzyme A ligase (4CL), and cinnamyl alcohol dehydrogenase (CAD) involved in the phenylpropane metabolism, enhancing the synthesis of the main precursors of suberin polyphenolic (SPP) and lignin, while transcriptional activation of the genes *StPOD* and *StNOX* and antimicrobial compounds, total phenolics, and flavonoids were also induced.

In rapeseed, Hu et al. demonstrated that both organic carbon (OC, 300 kg ha^-1^) and silicon (Si; 150 kg ha^-1^) applications increased the plant height, basal stem diameter, internode plumpness, and bending strength. Both OC and Si improved the activities of lignin biosynthesis enzymes (PAL, 4CL, CAD, and peroxiredoxins) and their related genes to increase lignin accumulation in the culm, which ultimately improved the lodging resistance. The thickness of the stem cortex, vascular bundle area, and xylem area were all increased, and the stem strength was improved. Silicon applications augmented the number of pods and yield, and improved the economic benefit.

In the model plant *Arabidopsis thaliana*, Pérez-Zavala et al. showed that Ti activates abscisic acid and salicylic acid signaling pathways and the expression of NUCLEOTIDE BINDING SITE-LEUCINE RICH REPEAT receptors likely by acting as a chemical priming molecule. This activation resulted in enhanced resistance to drought, high salinity, and infection with *Botrytis cinerea*. Ti also granted an enhanced nutritional state, even at suboptimal phosphate concentrations by upregulating the expression of multiple nutrient and membrane transporters and by modifying or increasing the production root exudates.

In rice, Anjum and Prakash applied three different sources of exogenous Si, namely diatomaceous earth (DE), silicic acid (SA) and rice husk biochar (RHB), and found that such Si sources significantly increased phytolith (Phy), phytolith occluded carbon (PhyOC) content, and readily soluble Si pools [acetic acid extractable Si (AASi), calcium chloride extractable Si (CCSi), and amorphous Si (ASi)] compared to the recommended dose of fertilizers (RDF: 100:50:50, N:P_2_O_5_:K_2_O kg ha^-1^), with the treatment receiving 4 t RHB ha^-1^ outperforming the other treatments. Phytolith and PhytOC production were found to be significantly correlated to soil organic carbon (OC), available N and K, and CCSi and ASi content in the soil. Treatments receiving 4 t RHB ha^-1^ have a stronger relationship with CCSi and ASi which majorly contributed to the higher phytolith and PhytOC production. Thus, practices such as Si and RHB application have a high potential for phytolith production and PhytOC sequestration, a critical mechanism of the global biogeochemical C sink.

In pedunculate oak, Kostic et al. studied the effect of Si supply on the leaf nutriome, root traits and overall growth of 12-week-old oak seedlings exposed to low P supply, biotic *Phytophthora plurivora* root infection, and their combination. The application of Si had the strongest ameliorative effect on growth, root health and root phenome under the most severe stress conditions. Silicon supply partially reversed the pattern of change of some leaf nutrients affected by stresses: P, B, and Mg under P deficiency, and P, B and S under pathogen attack, but also Ni and Mo under the combination of both factors.

**Figure 1:**
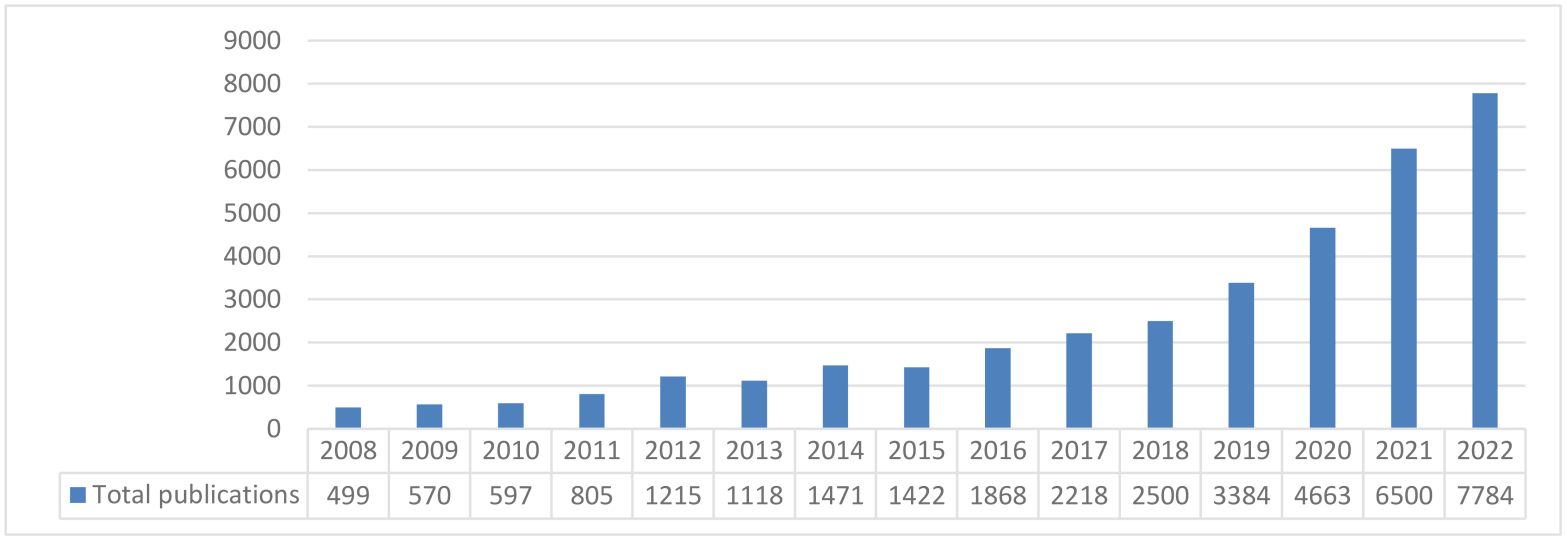
Evolution of the number of publications on the topic of “beneficial elements” during the last 15 years (2008-2022).

A bibliometric analysis of the literature over the last 15 years revealed that the number of publications on the 10 beneficial elements considered in the category of inorganic biostimulants has significantly grown, from nearly 500 in 2008, to 7784 in 2022 (**Figure 1**). This remarkable and progressive increase demonstrates once again the growing interest in this topic, from the scientific, technical and commercial perspectives.

## 4 Conclusions and perspectives

Biostimulation and particularly beneficial elements open up a new chapter in sustainable agriculture, which must be defined by a holistic approach. Importantly, biostimulants take a central stage in the worldwide push for green and regenerative agriculture, and represent an unstoppable global trend. The plant biostimulant market is projected to be $ 3.88 billion by 2023, which is expected to grow to $ 8.21 billion by 2030, with an annual growth rate of approximately 11%.

The main driving forces for the dynamic growth trend of the biostimulants market have been associated with a growing population demanding sufficient, nutritious, safer and innocuous foods that have to be produced in more restrictive agricultural environments exposed to more severe pressures imposed by global climate change (Pérez-Vázquez et al., 2018; Wijerathna-Yapa and Pathirana, 2022). In most modern societies, people are more aware of the impacts of agriculture on the environment, the value of nutraceutical foods to human health and the need to mitigate the effects of climate change on the global scenario. Therefore, there is a dire need to develop and implement a more efficient and effective use of the so-called “green chemicals” (Bhandari and Kasana, 2018; Fenibo et al., 2021; Ganesh et al., 2021), so that agricultural activities can cope with the increasing frequency of adverse environmental conditions for crop growth and productivity (Rouphael and Colla, 2020). In the coming years, the biostimulant market’s dynamics will be influenced by a more expanded regulatory framework, with beneficial elements playing a pivotal role in boosting or complementing the effects of other biostimulants such as amino acids, seaweed extracts and biofertilizers to achieve a modern and innovative crop production system worldwide. A second generation of biostimulants now exists, with specific biostimulatory action, a defined composition and synergic effects, to render agriculture more sustainable and resilient (Rouphael and Colla, 2018; Popa et al., 2022).

According to Ricci et al. (2019), it is the function of the product, not what it contains that defines a plant biostimulant. Consequently, demonstrating that a product is indeed a *bona fide* biostimulant depends on validation of its effect under different test conditions, which should not be misconstrued with ensuring efficacy. One must be aware that a commercial biostimulant may not guarantee effectiveness under all conditions, since many factors may influence its field performance.

Summing up, the studies included in this Research Topic describe the effects of the beneficial elements Na, Si and Ti boosting diverse mechanisms employed by plants to exhibit better performance under different environmental challenges. These studies shed light on how such elements may mediate or trigger tolerance responses in plants growing in restrictive conditions. Evidently, the research articles that are published in our Research Topic are of great importance, most of them being already cited only a few months after publication, showing the quality of the studies and also the interest given by readers and scientists for the respective reports. This digital Research Topic will make this issue more accessible to a broader range of readers.

It has been a great pleasure for us to write this foreword for such an inspiring, multi-authored international publication on a unique trending subject: inorganic biostimulation. We, therefore, express our sincere gratitude to all the contributors for their outstanding efforts in making this Research Topic a success.

## Author contributions

LIT-T: Investigation, Methodology, Resources, Validation, Writing – original draft, Writing – review & editing. FCG-M: Conceptualization, Funding acquisition, Methodology, Writing – original draft.
